# Anwendungen, Herausforderungen und ein vertrauenswürdiger Umgang mit künstlicher Intelligenz im Bereich Public Health

**DOI:** 10.1007/s00103-025-04098-2

**Published:** 2025-07-02

**Authors:** Joana Sarah Grah, Christopher Irrgang, Lars Schaade, Katharina Ladewig, Nils Körber

**Affiliations:** 1 Zentrum für Künstliche Intelligenz in der Public Health-Forschung, Robert Koch-Institut, Berlin, Deutschland; 2 Robert Koch-Institut, Berlin, Deutschland

**Keywords:** Künstliche Intelligenz, Public Health, Anwendungen, Daten, Vertrauenswürdige KI, Artificial intelligence, Public health, Applications, Data, Trustworthy AI

## Abstract

Künstliche Intelligenz (KI) hat sich in den letzten Jahren rasant weiterentwickelt und ist mittlerweile im Alltag der Bevölkerung angekommen. Durch die große Verfügbarkeit von vielfältigen Daten im Public-Health-Bereich ergibt sich eine Reihe von Anwendungsfeldern für KI. Diese reichen von der Infektionsforschung und Analyse epidemiologischer Daten über die Extraktion von Informationen aus Kommunikationsdaten wie sozialen Medien bis hin zur Entwicklung neuer Resilienzstrategien gegen den Klimawandel sowie der systematischen Auswertung von Fachliteratur.

Ausschlaggebend für den sinnvollen Einsatz von KI-Anwendungen sind die zugrunde liegenden Daten. In der Public-Health-Forschung gibt es auf der einen Seite eine große Variabilität von Datentypen, bspw. Bilddaten, numerische Daten, Umfragedaten u. v. m. Andererseits ist die Datenverfügbarkeit oftmals gering, z. B. wenn eine seltene Pathologie untersucht und/oder hohe Datenschutzanforderungen gestellt werden. Gleichzeitig müssen hohe ethische Standards erfüllt werden und Verzerrungen, Unausgewogenheiten und Intransparenz möglichst früh erkannt und minimiert werden.

Wir zeigen einen möglichen Weg zu einem verantwortungs- und vertrauensvollen Umgang mit KI-Anwendungen im Public-Health-Bereich, der von der Fragestellung über Daten und Modell zur Evaluation führt und die Wichtigkeit einer sorgfältigen und vollständigen Dokumentation hervorhebt.

## Abstract (en)

The rapid advancements in artificial intelligence (AI) over recent years have resulted in its integration into people’s everyday lives. The wide availability of diverse data within the public health sector opens up a number of fields of application for AI, ranging from infection research and the analysis of epidemiological data to the extraction of information from communication data such as social media, the development of new resilience strategies against climate change and the systematic evaluation of specialist literature.

The quality of the underlying data is paramount to the successful implementation of AI applications. In public health research, on the one hand, there is a wide variability of data types including, but not limited to, image data, numerical data and survey data. On the other hand, availability can be limited, for example when a rare pathology is being investigated and/or stringent data protection requirements apply. Concurrently, it is imperative to maintain high ethical standards and to mitigate biases, imbalances and lack of transparency as early as possible.

We delineate an approach towards the responsible and trustworthy utilisation of AI applications in public health, which leads from the initial question to the data and the model development to evaluation and emphasises the importance of careful and complete documentation.

Die Anwendung von künstlicher Intelligenz (KI) hat Einzug in viele Bereiche des täglichen Lebens gehalten und hat das Potenzial, die Gesundheit und Gesundheitsversorgung der Bevölkerung grundlegend zu verbessern. Während KI-Anwendungen in der medizinischen Diagnostik und Therapie schon länger erforscht und auch schrittweise in die Praxis überführt werden [1, 2], ist die Implementation im Public-Health-Bereich noch im Anfangsstadium. In den letzten 10–15 Jahren haben sich vor allem Deep-Learning-Methoden rasant weiterentwickelt. War deren theoretische Basis schon seit der zweiten Hälfte des letzten Jahrhunderts vorhanden [3–5], so konnten sie erst mit der exponentiell wachsenden Menge von Daten und der Verbesserung von Computer-Hardware erfolgreich eingesetzt werden. Zunächst wurden sogenannte Convolutional Neural Networks speziell im Bereich der Bildverarbeitung sehr populär und erzielten auch in der medizinischen Bildgebung beispielsweise für Rekonstruktion, Klassifikation und Objekterkennung hervorragende Ergebnisse. Während sich für die Analyse von Bildern und Texten zunächst unterschiedliche Modelle durchsetzten, wurde mit der Entwicklung der Transformer-Modelle [6], auf der auch alle aktuellen großen Sprachmodelle basieren, eine leistungsstarke einheitliche Modellarchitektur geschaffen. Durch die einheitliche Verarbeitung kommen vermehrt multimodale Modelle zum Einsatz, die Informationen aus verschiedenen Quellen und Typen verarbeiten und verknüpfen können. Aufgrund der historischen Entwicklung der Modelle wird der Begriff KI für eine Bandbreite von Methoden verwendet. Im Folgenden verwenden wir den Begriff KI für alle datengetriebenen Methoden, die mithilfe des maschinellen Lernens Muster erkennen und Vorhersagen treffen. Dabei kann es sich um klassische Verfahren wie Random Forests oder Deep-Learning-Modelle bis hin zu großen Sprachmodellen mit Milliarden von Parametern handeln.

KI-Methoden haben Einzug in nahezu alle wissenschaftlichen Disziplinen gehalten und zu großen Fortschritten, wie zum Beispiel in der Proteinfaltung, geführt [7]. Die Public-Health-Forschung ist traditionell eine datengetriebene Wissenschaft und dadurch ein vielversprechendes Anwendungsfeld für KI-Verfahren. Wir beschäftigen uns in dieser Arbeit mit der Frage, welche Potenziale KI im Public-Health-Bereich bietet, welche Risiken damit einhergehen und wie ein vertrauenswürdiger Umgang für Anwendungen geschaffen werden kann.

Im Folgenden gehen wir auf Anwendungen von KI im Public-Health-Bereich ein, die von der Erregercharakterisierung und Ausbruchserkennung über die epidemiologische Modellierung bis zur Evidenzerzeugung anhand der Analyse großer Mengen wissenschaftlicher Literatur reichen. Darüber hinaus kann KI helfen, Gesundheitsrisiken verursacht durch den Klimawandel zu erkennen und eine gezielte Kommunikation in einer Informationsflut zu ermöglichen. Im zweiten Abschnitt diskutieren wir Public-Health-Daten sowie damit einhergehende Herausforderungen. Die Aussagefähigkeit aller KI-Modelle ist in großem Maße von den zugrunde liegenden Daten abhängig. Die Qualität der Trainingsdaten steht damit im Mittelpunkt der KI-Entwicklung. Dabei ist es essenziell, Unausgewogenheiten in den Daten frühzeitig zu erkennen und in der Implementierung zu berücksichtigen, um unproportionale Vorhersagen und Diskriminierung zu reduzieren. Vor allem für gesundheitsrelevante Anwendungen sind die Auswirkungen groß, sodass bei der Integration in Fachverfahren große Sorgfalt notwendig ist. Die bestehenden gesetzlichen Regelungen sollten mit einem verantwortungswürdigen und transparenten Umgang mit KI einhergehen. Einen Weg für die Entwicklung solcher KI-Modelle zeigen wir im letzten Abschnitt auf.

## Anwendungen von KI im Bereich Public Health

**Abb. 1:**
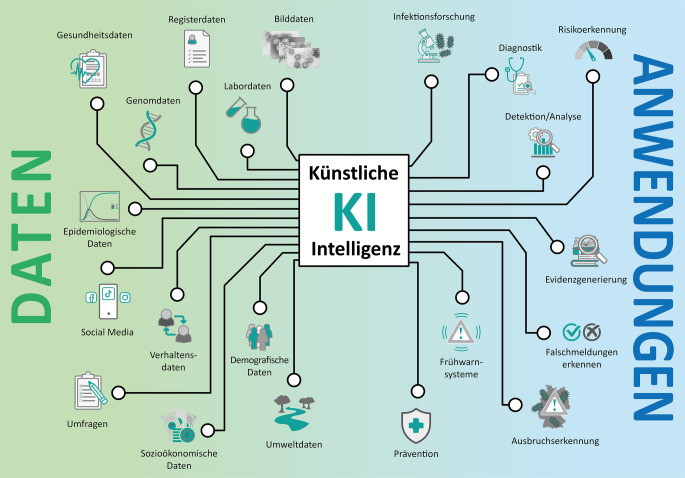
Daten und Anwendungen von künstlicher Intelligenz (KI) im Bereich Public Health. (Quelle: eigene Abbildung)

Durch die Fähigkeit, große Datenmengen schnell zu analysieren und komplexe Muster zu erkennen, eröffnet KI neue Möglichkeiten in der Automatisierung und Untersuchung gesundheitsrelevanter Daten. Von der Evidenzerzeugung bis hin zur Vorhersage von Krankheitsausbrüchen können KI-basierte Anwendungen dabei unterstützen, die öffentliche Gesundheit zu stärken und zu schützen (Abb. 1). Wir geben im Folgenden einen Überblick über die Vielzahl verschiedener Einsatzmöglichkeiten von KI im Bereich Public Health.

Beginnen wir mit einem Blick auf die Infektionsforschung und Erregercharakterisierung, in denen der Einsatz von KI neue Perspektiven eröffnet. Durch die Analyse großer genomischer Datensätze können KI-Algorithmen relevante Mutationen identifizieren, die für die Entstehung neuer Virusvarianten oder die Ausbreitung von Infektionen verantwortlich sind [8, 9]. Diese Erkenntnisse sind entscheidend für die Untersuchung von Ausbrüchen, die Überwachung der Verbreitung verschiedener Virusvarianten und die Etablierung gezielter Interventionsmaßnahmen. Die Entwicklung datengetriebener diagnostischer Verfahren auf Basis von KI-Modellen erweitert das Methodenspektrum der Erregererkennung [10]. Moderne bildgebende Verfahren erzeugen in kurzer Zeit große Datenmengen. KI-Verfahren können dabei helfen, die Analyse zu automatisieren, schwer zu erkennende Muster detektieren sowie den manuellen Aufwand reduzieren und so zu quantitativen Ergebnissen führen. Beispielsweise können KI-Systeme verschiedene Erreger morphologisch charakterisieren und so eine schnelle und genaue Diagnose ermöglichen [11]. Andererseits können KI-Modelle dabei helfen, Muster zu unterscheiden, die manuell nur schwer zu differenzieren sind, wie beispielsweise die Unterscheidung verschiedener Virusinfektionen [12].

Gehen wir einen Schritt von der Erreger- auf die Populationsebene, so ergibt sich eine Vielfalt von KI-Anwendungsfällen im Bereich der Epidemiologie. Die Modellierung von epidemiologischen Daten, die Früherkennung von Krankheitsausbrüchen und die Vorhersage der Krankheitslast sind essenziell für den Bevölkerungsschutz. Die Covid-19-Pandemie hat dem Forschungsfeld der epidemiologischen Modellierung einen enormen Schub versetzt, wobei mechanistische und statistische Modelle zunehmend durch KI-Modelle ergänzt wurden [13]. Während mechanistische Modelle den kausalen Infektionszyklus berücksichtigen und eine hohe Nachvollziehbarkeit aufweisen, basieren die Vorhersagen von KI-Modellen auf den verwendeten Daten und können auch in der komplexen Dynamik der realen Welt Zusammenhänge erkennen [14]. Darüber hinaus ermöglichen KI-Modelle die Verwendung und Kombination von strukturierten und unstrukturierten Daten aus unterschiedlichen Quellen. Durch die Verwendung von hybriden Modellen wird versucht, das Domänenwissen von mechanistischen Modellen mit der Mustererkennung von KI-Methoden zu vereinen.

Die Informationsbeschaffung der Bevölkerung unterliegt einem steten Wandel, der aktuell vor allem durch soziale Medien getrieben ist. Die Verbreitung von Falschinformationen zu gesundheitlich relevanten Themen ist dabei eine Gefahr für die Akzeptanz von gesundheitlichen Schutzmaßnahmen. Im Zuge der Covid-19-Pandemie kam es durch die Informationsflut und die uneingeschränkte Verbreitung dieser Informationen im Internet und in den sozialen Medien zu einer weitreichenden Verbreitung von Falschmeldungen und Verschwörungserzählungen, wodurch das Gesundheitsverhalten maßgeblich beeinflusst wurde. In diesem Zusammenhang wurde der Begriff der Infodemie geprägt [15, 16]. KI kann dabei helfen, den Diskurs in sozialen Medien zu analysieren, und so eine zielgerichtete Kommunikation ermöglichen [17]. Darüber hinaus können mithilfe von KI-Verfahren große Mengen von Beiträgen aus sozialen Medien auf Falschmeldungen untersucht werden [18]. Durch die Identifikation von Falschmeldungen können eine zielgerichtete Kommunikation und korrekte Faktendarstellung erreicht werden. Besonders gesundheitsrelevante Falschinformationen können gravierende Auswirkungen bei der Bekämpfung des Infektionsgeschehens haben.

Eine der größten Herausforderungen für die öffentliche Gesundheit in den kommenden Jahren ist die Auswirkung des anthropogenen Klimawandels [19, 20]. Die Gesundheitsrisiken können dabei ganz unterschiedlicher Natur sein. Als direkte Folge wird die erhöhte Oberflächentemperatur zu einer Zunahme der hitzebedingten Mortalität führen. KI-Methoden können dabei helfen, hochaufgelöst Risikogebiete zu identifizieren und präventive Maßnahmen auf kommunaler Ebene zu empfehlen [21]. Neben den direkten Auswirkungen gibt es auch indirekte Folgen. So breiten sich invasive Arten wie die asiatische Tigermücke *Aedes albopictus* infolge veränderter klimatischer und ökologischer Bedingungen auch in Mittel- und Nordeuropa aus [22]. Dies ermöglicht die autochthone Übertragung von Arboviren wie dem Zika‑, Chikungunya- und Dengue-Virus. KI-Methoden in Kombination mit Klimasimulationen können dazu verwendet werden, potenzielle Risikogebiete für arbovirale Ausbrüche zu identifizieren [23]. Insbesondere die Kombination der Vorhersage von Risikogebieten mit epidemiologischen Modellen stellt eine vielversprechende Perspektive zur Bekämpfung von vektorübertragenen Krankheiten dar.

In der Public-Health-Forschung gelten Metaanalysen und systematische Reviews als Goldstandard zur Evidenzerzeugung. Angesichts der exponentiell wachsenden Zahl wissenschaftlicher Publikationen stößt die manuelle Auswertung jedoch zunehmend an ihre Grenzen. Obwohl auch die neuesten großen Sprachmodelle noch nicht in der Lage sind, wissenschaftliche Texte fehlerfrei zu analysieren, bietet Natural Language Processing (NLP) ein großes Potenzial, den manuellen Aufwand bei der Informationsextraktion deutlich zu reduzieren. So können beispielsweise Klassifikationsmodelle trainiert werden, relevante von irrelevanten Studien zu unterscheiden, wodurch das Screening erheblich vereinfacht wird [24]. Darüber hinaus können alle Phasen eines systematischen Reviews – von der Literatursuche über die Selektion und Datenextraktion bis hin zur Qualitätsbewertung und Datenzusammenführung – durch maschinelles Lernen unterstützt werden. Eine umfassende Übersicht über verfügbare Softwarelösungen für die einzelnen Schritte bietet ein 2022 veröffentlichtes Review von Cierco Jimenez et al. [25]. Durch den Einsatz von KI kann die Effizienz systematischer Reviews signifikant gesteigert werden. Dennoch sollte die menschliche Expertise weiterhin eine zentrale Rolle spielen, um die Transparenz, Nachvollziehbarkeit und Vertrauenswürdigkeit der Ergebnisse zu gewährleisten. Eine Kombination aus menschlicher Beurteilung und algorithmischer Unterstützung ist daher der vielversprechendste Weg, um die Qualität von Evidenzsynthesen zu sichern.

Zusammenfassend können KI-Methoden im gesamten Spektrum der Public-Health-Forschung eingesetzt werden. Vorteile ergeben sich vor allem in Bereichen, in denen viele qualitätsgesicherte Daten vorliegen. KI-Verfahren können dann eingesetzt werden, um Abläufe zu vereinfachen und auch sehr komplexe Zusammenhänge zu erkennen, die mit klassischen statistischen Verfahren bisher nicht erfassbar waren. Um transparente und vertrauenswürdige KI-Verfahren zu entwickeln, sollte jedoch menschliche Aufsicht in alle Teilschritte mit einbezogen werden. Ein besonderer Fokus liegt dabei auf den Trainingsdaten, die die Grundlage für jedes KI-Verfahren bilden und somit auch entscheidend für die Qualität der Modelle sind.

## Public-Health-Daten

In Abb. 1 sehen wir, wie vielfältig Public-Health-relevante Daten sein können: Biomedizinische Bilddaten beinhalten z. B. mikroskopische Bilder, etwa von einem Licht- oder Elektronenmikroskop, oder medizinische Aufnahmen von Röntgen‑, Computertomographie-, Magnetresonanztomographie- oder Ultraschallgeräten. Genomdaten können sich auf menschliche DNA beziehen oder auch Aufschluss über die Struktur und Funktion von Pathogenen geben. Während der Covid-19-Pandemie spielten insbesondere epidemiologische Daten eine tragende Rolle: Inzidenzen, Reproduktionszahlen und das Verhältnis zwischen (vereinfacht) Infizierbaren (*Susceptibles*; S), Infizierten (*Infected*; I) und Genesenen (*Recovered*; R) in SIR-Modellen sind vielen Menschen mittlerweile ein Begriff. Weitere numerische Datentypen im Gesundheitswesen sind Labor‑, Register‑, Gesundheits- und demografische Daten. In Ergänzung dazu gibt es auch aufschlussreiche qualitative Daten, die beispielsweise durch Umfragen gewonnen werden. Eine immer zugänglicher werdende Klasse von Daten sind Verhaltensdaten, die z. B. die Nutzung von Website-Inhalten oder Apps analysieren. Dabei spielt insbesondere die Nutzung von sozialen Medien eine immer bedeutendere Rolle. Zusätzlich bilden Verhältnisdaten wie Umwelt- und sozioökonomische Daten eine wichtige Grundlage.

Sind personenbezogene Informationen enthalten, sind die Daten besonders sensibel und unterliegen Datenschutzvorschriften. In Deutschland greift das Bundesdatenschutzgesetz und EU-weit die Datenschutzgrundverordnung (DSGVO). Daraus resultiert eine oft sehr begrenzte Verfügbarkeit der personenbezogenen Daten. Im Vergleich zu anderen inner- und außereuropäischen Ländern wird der Datenschutz in Deutschland in der Praxis besonders streng ausgelegt und so wird seit einiger Zeit eine öffentliche Debatte darüber geführt, ob dies die Entwicklung und vor allem Wettbewerbsfähigkeit im Bereich KI hemmt. Um die Datengrundlage für KI-Methoden zu erhöhen, gibt es aber einige Lösungsansätze. Zum einen gibt es mittlerweile Techniken zur verlässlichen Anonymisierung von Daten, die die Aussagekraft von Modellen, die auf diesen Daten trainiert werden, nicht oder nur in geringem Maße reduzieren [26]. Zum anderen kann generative KI eingesetzt werden, um die Datenmenge zu erhöhen [27]. Überdies ist Datenerweiterung (engl. Data Augmentation) eine gängige Praxis in der Implementierung von KI-Methoden, um den Input zu vergrößern, indem z. B. bei Bilddaten gespiegelte, rotierte oder vergrößerte Versionen der Originalbilder mit in das Modell fließen [28].

**Abb. 2:**
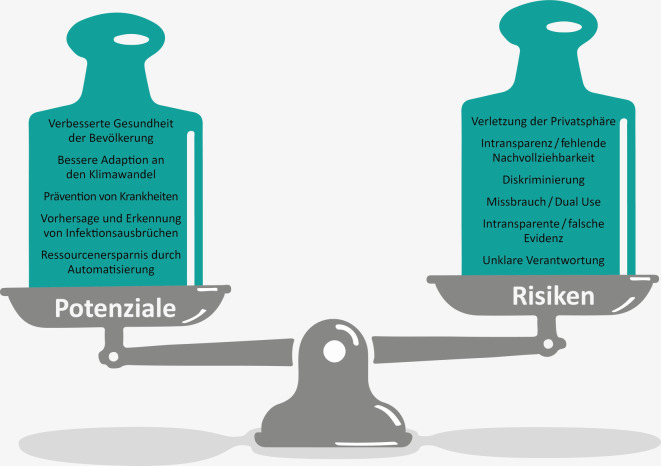
Potenziale und Risiken von KI-Anwendungen im Bereich Public Health. (Quelle: eigene Abbildung)

Die Entwicklung von KI birgt vielversprechende Potenziale für das Gesundheitswesen, allerdings gehen damit auch große Risiken durch falsche oder missbräuchliche Nutzung einher (Abb. 2). Im Jahr 2019 wurde von der Europäischen Kommission eine Ethikleitlinie für eine vertrauenswürdige KI veröffentlicht^1^ und 2020 eine darauf basierende Liste zur Selbsteinschätzung für KI-Entwickler*innen und andere Akteur*innen^2^. Die sieben identifizierten Kernanforderungen wollen wir im Folgenden vor dem Hintergrund des Gesundheitswesens diskutieren.

*Der Vorrang menschlichen Handelns und die menschliche Aufsicht* sind gerade im Public-Health-Bereich elementar. Vor allem bei medizinischen Anwendungen sollten Ärzt*innen immer die Letztentscheidung treffen, wenn es um KI-gestützte Diagnosen oder Therapievorschläge geht. *Technische Robustheit und Sicherheit* spielen eine ähnlich entscheidende Rolle, gerade im Umgang mit sensiblen Daten. Die IT-Infrastruktur sollte hohen Sicherheitsstandards unterliegen und Datenspeicherung und -transfer sollten ausschließlich verschlüsselt erfolgen. Auch vor Datenmanipulation, wie etwa sogenannten Adversarial Attacks, sollte ein hoher Schutz bestehen. In einer Arbeit von Finlayson et al. [29] wird z. B. demonstriert, dass Bilder aus der Dermatologie so manipuliert werden können, dass sie optisch nicht vom Original zu unterscheiden sind, aber sich eine Diagnose dadurch von „gutartig“ zu „bösartig“ oder umgekehrt ändern kann. Ein Beispiel aus der natürlichen Sprachverarbeitung zeigt, dass manipulative Textsubstitution zu falschen Risikobewertungen bzgl. des Missbrauchs von Opioiden führen kann. *Datenschutz und Datenqualitätsmanagement* gehen einerseits mit technischer Sicherheit einher und umfassen andererseits die Anonymisierung bzw. Pseudonymisierung von personenbezogenen Daten. Vor allem bei gesundheitsbezogenen Daten ist der Schutz individueller Informationen von größter Wichtigkeit, um das Vertrauen in den Einsatz von KI nicht nachhaltig zu beschädigen. Eine weitere wichtige Kernanforderung ist die *Transparenz* [30]. Dazu zählt nicht nur das sich rapide entwickelnde Feld der erklärbaren KI (engl. Explainable AI, XAI; [31]), in dem es darum geht, Vorhersagen von KI-Modellen für Anwender*innen verständlich und nachvollziehbar zu machen, sondern auch die Reproduzierbarkeit von Ergebnissen und sorgfältige Dokumentation (siehe z. B. [32] für Daten- und [33] für Modelldokumentation). Die *Vielfalt, Nichtdiskriminierung und Fairness* sind eng verbunden mit den Daten eines KI-Modells und damit aber auch Teil der Verantwortung der Entwickler*innen. KI-Modelle bilden immer die zugrunde liegenden Daten ab, die ggf. auch die vorherrschenden gesellschaftlichen Verhältnisse und Werte widerspiegeln. Soziale Ungleichheit etwa spiegelt sich auch in der Gesundheit wider [34] und kann durch Anwendung von KI-Methoden verstärkt werden. Verantwortlich dafür sind Bias, d. h. Verzerrungen und Voreingenommenheiten, die aus den Daten hervorgehen. Diese Bias in den Trainingsdaten können Rassismus, Sexismus, Homophobie und andere Formen von Diskriminierung verstärken. Daher ist es essenziell, dass Daten auf mögliche Ungleichgewichte und Bias hin geprüft und ggf. korrigiert werden. Ganz eliminieren lassen sie sich allerdings nicht, aber die Bewusstmachung und Reflexion sind hier entscheidend und in die Schlussfolgerung der Analyse mit einzubeziehen. Eine Unausgewogenheit in den Daten ist nicht nur zur Vermeidung von Diskriminierung relevant, beispielsweise könnte auch ein Krankheitstyp in den Daten unterrepräsentiert und die Vorhersagen des entsprechenden Modells dazu deshalb weniger genau sein. Eine weitere Kernanforderung für vertrauenswürdige KI ist *das gesellschaftliche und ökologische Wohlergehen*. Auf der einen Seite ist bekannt, dass gerade das Trainieren und Anwenden von Deep-Learning-Methoden mit großen Datenmengen und komplexen Modellen mit einem hohen Energieverbrauch einhergehen [35, 36]. Andererseits gibt es ein breites Forschungsfeld zur Klimaanalytik, das sich KI-Methoden zunutze macht. Letztlich ist die *Rechenschaftspflicht,* wie im Kontext der Letztentscheidung schon erwähnt, im Gesundheitswesen elementar [37, 38].

Die gesetzliche Regulierung von KI-Methoden im Public-Health-Bereich beschränkt sich keineswegs auf den Datenschutz. Im August 2024 ist der „AI Act“, das EU-Gesetz zur KI, in Kraft getreten.^3^^,^^4^ Kern ist der risikobasierte Ansatz, nach dem Anwendungen in KI-Systeme mit minimalem Risiko, besonderen Transparenzverpflichtungen, hohem Risiko und unannehmbarem Risiko eingestuft werden. KI-basierte Medizinprodukte, wie z. B. KI-Systeme zur Unterstützung klinischer Entscheidungen, bergen ein hohes Risiko und unterliegen nach dem AI Act strengen Anforderungen. Ein KI-Chatbot, der medizinische Empfehlungen gibt oder zur psychotherapeutischen Interaktion eingesetzt wird, unterliegt neben den Anforderungen für Medizinprodukte auch den besonderen Transparenzverpflichtungen des AI Act [39]. Die Regularien und auch Verbote sind eng verknüpft mit Grundrechten und -werten von Menschen und haben u. a. zum Ziel, eine bessere Gesundheitsversorgung und effizientere öffentliche Dienste für Bürger*innen zu ermöglichen. Vorhaben, deren einziger Zweck in Forschung und Entwicklung liegt, sind von der KI-Verordnung ausgenommen.

Insbesondere überwachte Lernmethoden erfordern das Labeln, also das Markieren, von Daten. Das bedeutet, dass im Falle einer Klassifizierung z. B. positive und negative Beispiele gekennzeichnet werden. In Public-Health-Anwendungen ist das Labeln tendenziell sehr aufwendig und oft nur mit Domain-Expertise durchführbar. Die Qualität der Daten hat einen direkten Einfluss auf die Resultate der Modelle, von daher sollte besondere Sorgfalt auf die Auswahl der Trainingsdaten gelegt werden. Um fehlende oder unvollständige Daten zu kompensieren, müssen Techniken wie Datenerweiterung, -imputation (also das Einsetzen von statistisch passenden Daten an Stellen, wo Daten fehlen), Transfer Learning mit auf großen Datensätzen vortrainierten Modellen oder teil- bzw. unüberwachte Lernmethoden angewandt werden. Oft unterliegen Daten im Gesundheitswesen einer ungleichen Verteilung, wenn es z. B. um das Auftreten von seltenen Krankheiten oder eine fehlende Repräsentation von Geschlechtern oder Altersgruppen in den Daten geht. Auch in diesem Fall kann eine Erweiterung von weniger repräsentierten Daten, Stratifizierung oder die Generierung von (synthetischen) Daten helfen. Außerdem kann die Unausgewogenheit in die Optimierung des Modells einfließen.

## Der Weg zu vertrauenswürdiger KI

**Abb. 3:**
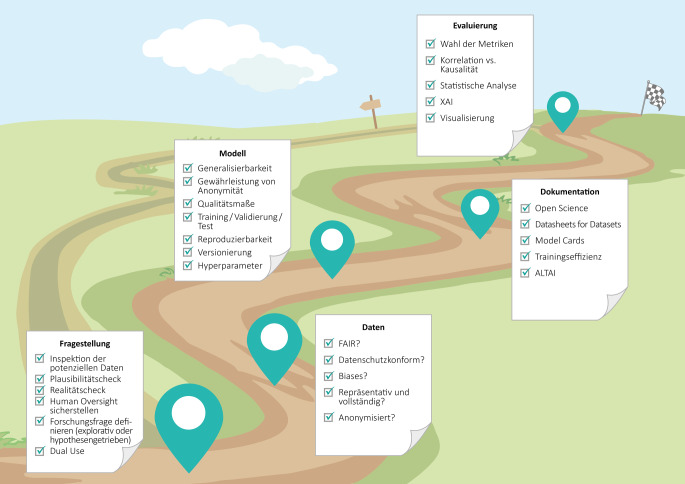
Ein Weg zu vertrauenswürdiger KI. FAIR – Findable, Accessible, Interoperable, Reusable; XAI – Explainable AI; ALTAI – Assessment List for Trustworthy AI. (Quelle: eigene Abbildung)

Während es zahlreiche Anwendungsfälle und erfolgreiche Beispiele in der Medizin und Public-Health-Forschung gibt, fehlt ein standardisierter Weg, um transparente, vertrauenswürdige Modelle zu schaffen [40]. Hier zeigen wir einen verantwortungsvollen und mit wissenschaftlichen Werten zu vereinbarenden Weg der Entwicklung und Implementierung einer KI-Anwendung im Gesundheitswesen, der sich natürlich auch auf andere Anwendungen übertragen lässt (Abb. 3). Am Anfang steht das Problem bzw. die zu beantwortende Fragestellung. Dabei kann es sich um datengetriebene (explorative) oder hypothesengetriebene Forschung handeln. Darüber hinaus werden KI-Modelle häufig als Methode zur Auswertung von großen Datenmengen verwendet. Für eine gute wissenschaftliche Praxis ist es wichtig, zu Beginn die Hypothese oder die zu erreichenden Zielmetriken zu definieren, die am Ende des Weges validiert, widerlegt oder erreicht werden sollen, um sogenanntes HARKing (Hypothesis After Results are Known; [41]) zu vermeiden. Dieses sollte allerdings nicht die erste Inspektion von potenziellen Daten beeinflussen (siehe [42]), die unvoreingenommen erfolgen sollte. Zusätzlich sollte der gesamte Projektverlauf vorläufig geplant und einem Plausibilitäts- sowie Realitätscheck unterzogen werden. Bei medizinischen Anwendungen sollte sichergestellt werden, dass die Studie finanziell, personell und mit den vorhandenen Ressourcen durchführbar ist. Außerdem müssen bei epidemiologischen Studien genug Personen ihr Einverständnis zur Teilnahme und Datenverarbeitung geben. In vielen Fällen ist ein Ethikvotum notwendig und die Verarbeitung, Speicherung und ggf. notwendige Weitergabe der Daten sollten vorab geregelt werden. Darüber hinaus sollte bereits zu Beginn eines Projektes das potenzielle Missbrauchsrisiko (Dual Use) der KI evaluiert und ggf. Gegenmaßnahmen wie beschränkter Zugang getroffen werden.

Der nächste Schritt auf dem Weg zu einer fairen und vertrauenswürdigen KI ist die Vorbereitung der Daten. Sie sollten die FAIR-Prinzipien [43] erfüllen und außerdem repräsentativ und vollständig sein. Darüber hinaus muss die Datenverarbeitung datenschutzkonform gemäß DSGVO erfolgen. Die Daten sollten auf potenzielle systematische Fehler und Verzerrungen und daraus resultierende Diskriminierung und Benachteiligung geprüft und gegebenenfalls überarbeitet werden. Auch hier ist zu beachten, dass eine Diversität der Daten gewährleistet ist, also dass etwa soziodemografische Merkmale idealerweise nahezu gleichverteilt sind oder dass bei selteneren Pathologien auch ausreichend Beispiele des Krankheitsbildes vorliegen. Sind die Daten personenbezogen bzw. besteht die Gefahr der Verletzung der Privatsphäre, sollten sie anonymisiert verarbeitet werden, auch um die Daten vor sogenannten Einschleusungs- bzw. Injektionsangriffen (engl. Injection Attacks) zu schützen. Clusmann et al. haben z. B. untersucht, ob die aus einer Bildgebung gewonnene Diagnose, wie etwa das Vorhandensein oder die Abwesenheit eines Tumors, nach Belieben manipuliert werden kann, indem bösartige Informationen in den Modelleingabetext eingespeist werden [44].

Mit den vorbereiteten Daten kann nun die Analyse durchgeführt werden. Eine saubere Aufteilung in Trainings‑, Validierungs- und Testdaten ist für KI-Anwendungen gängige Praxis, um die Aussagekraft des Modells beurteilen zu können. Die 3 Teile des Datensatzes, der analysiert werden soll, sollten aus unabhängigen Stichproben sein und dürfen sich nicht überschneiden. Niemals dürfen Testdaten vor Abschluss der Analyse verwendet werden, um ein Modell zu evaluieren. Die statistischen Eigenschaften der Testdaten, nicht zwingend der Trainingsdaten, sollten dabei mit denen der realen Anwendung übereinstimmen und zur Analyse etwaiger Bias und Ungleichgewichte verwendet werden. Ein großer Vorteil datengetriebener KI-Methoden gegenüber klassischen Methoden ist die Generalisierbarkeit, also die Übertragbarkeit auf einen ähnlichen Kontext.

Zur guten wissenschaftlichen Praxis zählt auch eine vollständige und schlüssige Dokumentation. Zur besseren Nachvollziehbarkeit empfiehlt es sich, nicht nur Programmcode zu versionieren, sondern auch einzelne Trainingsläufe mit zugehörigen Hyperparametern und verwendeten Trainingsdaten. Im Bereich der KI-Forschung ist der Open-Science-Gedanke weitverbreitet und es werden zunehmend neben der wissenschaftlichen Publikation und dem zugehörigen Programmcode zum Reproduzieren der Ergebnisse auch die zugrunde liegenden Datensätze veröffentlicht. Nicht zuletzt während der Covid-19-Pandemie hat sich gezeigt, wie wichtig ein schneller und transparenter Austausch von Studienergebnissen zum Beispiel über Preprint-Server ist, um auf wissenschaftliche Artikel bereits vor der finalen Veröffentlichung zugreifen zu können. Mithilfe der von der Europäischen Kommission veröffentlichten Assessment List for Trustworthy Artificial Intelligence (ALTAI) kann geprüft werden, ob es ethische Fragen oder Probleme gibt. Nicht zuletzt sollte vor dem Hintergrund des Klimawandels auch die Trainingseffizienz berücksichtigt werden und es sollten unnötige Trainingsläufe vermieden werden.

Ähnlich wie die Wahl des Fehlermaßes während der Analyse hat auch die Wahl der Evaluationsmetrik einen großen Einfluss auf die Auswertung. Sie wird abhängig davon gewählt, ob sich damit die Fragestellung beantworten bzw. die Hypothese bestätigen oder widerlegen lässt. In diesem Sinne ist die Wahl der Evaluationsmetrik subjektiv, da sie bestimmt, welches Ergebnis als gut bewertet wird. Oft gibt es mehrere geeignete Metriken für eine Problemstellung. Die Metrik(en) sowie ein zu erreichender Zielwert sollten schon zu Beginn des Projektes festgelegt werden. Die Evaluation kann sowohl quantitativ als auch qualitativ erfolgen. Bei den meisten KI-Anwendungen geht es darum, einen bestimmten Wert einer Qualitätsmetrik zu erlangen oder zu übersteigen. Es können weitere statistische Analysen folgen, um ein besseres Verständnis der Ergebnisse zu erlangen. Wann immer möglich, sollten Modelle auch auf Plausibilität getestet werden. Die Kombination mit mechanistischen Modellen kann die Modelle robuster machen, insbesondere wenn nur wenige Trainingsdaten vorliegen. Auch XAI kann hier zum Einsatz kommen, um die Ergebnisse besser interpretieren zu können. Ein ganzes Forschungsfeld der KI beschäftigt sich außerdem mit der sinnvollen und verständlichen Visualisierung von Ergebnissen. Bei allen Schlussfolgerungen ist jedoch immer zu unterscheiden, ob es sich um Korrelationen oder Kausalitäten handelt. Wird ein Modell wiederholt mit unterschiedlichen Daten verwendet, sollte zudem auch die Evaluation kontinuierlich wiederholt werden. In der Praxis wird häufig ein sogenannter Drift beobachtet, d. h. eine Abnahme der Vorhersagegenauigkeit aufgrund der sich zeitlich ändernden statistischen Eigenschaften der Eingabedaten.

Zusammenfassend können KI-Anwendungen universell für Public-Health-relevante Fragestellungen verwendet werden und insgesamt dazu beitragen, die Gesundheit der Bevölkerung zu verbessern. Allerdings sollten wie bei allen wissenschaftlichen Arbeiten systematisch Fehler analysiert und möglichst vermieden werden. Dazu ist eine umfassende Analyse der zugrunde liegenden Daten und trainierten Modelle unbedingt notwendig.

## Footnotes

1 High-level expert group on artificial intelligence (2019) Ethics guidelines for trustworthy AI. European Commission. https://digital-strategy.ec.europa.eu/en/library/ethics-guidelines-trustworthy-ai. Zugegriffen: 17.01.2025.

2 High-level expert group on artificial intelligence (2020) Assessment List for Trustworthy Artificial Intelligence (ALTAI) for self-assessment. European Commission. https://digital-strategy.ec.europa.eu/en/library/assessment-list-trustworthy-artificial-intelligence-altai-self-assessment. Zugegriffen: 17.01.2025.

3 Generaldirektion Kommunikation (2024) KI-Verordnung tritt in Kraft. Europäische Kommission. https://commission.europa.eu/news/ai-act-enters-force-2024-08-01_de#:~:text=Am%201.,%2DVerordnung)%20in%20Kraft%20getreten. Zugegriffen: 17.01.2025.

4 Verordnung (EU) 2024/1689 des Europäischen Parlaments und des Rates vom 13. Juni 2024 zur Festlegung harmonisierter Vorschriften für künstliche Intelligenz und zur Änderung der Verordnungen (EG) Nr. 300/2008, (EU) Nr. 167/2013, (EU) Nr. 168/2013, (EU) 2018/858, (EU) 2018/1139 und (EU) 2019/2144 sowie der Richtlinien 2014/90/EU, (EU) 2016/797 und (EU) 2020/1828 (Verordnung über künstliche Intelligenz) (2024) OJ L. https://eur-lex.europa.eu/legal-content/DE/TXT/HTML/?uri=OJ:L_202401689. Zugegriffen: 17.01.2025.
